# Mastectomy rates remain high in Singapore and are not associated with poorer survival after adjusting for age

**DOI:** 10.1186/s40064-015-1460-2

**Published:** 2015-11-10

**Authors:** Patrick M. Y. Chan, Bok Ai Choo, Tianjiao Zhang, Melanie D. W. Seah, Juliana J. C. Chen, Sarah Q. H. Lu, Ern Yu Tan

**Affiliations:** Department of General Surgery, Tan Tock Seng Hospital, 11 Jalan Tan Tock Seng, Singapore, 308433 Singapore; Department of Radiation Oncology, National University Cancer Institute, Singapore, Singapore

**Keywords:** Mastectomy, Wide local excision, Overall survival

## Abstract

Recent reports have suggested that women undergoing mastectomy, instead of wide local excision (WLE) for Stage I and II breast cancers have poorer overall survival. This is particularly important in our setting where mastectomy rates are high. In this study, we evaluated the trends in mastectomy and WLE over a 10-year period at a single institute, identified factors more common among women who underwent mastectomy and specifically examined the effect of surgery on outcome. Retrospective review was performed of 2244 women who underwent curative surgery for non-metastatic breast cancer at our institute from 1st January 2001 to 31st December 2010. Mastectomy rates remained high over the 10 years, ranging from 43 to 59 %. Older women, those with symptoms, larger tumours and clinical nodal involvement were more likely to receive mastectomy (P < 0.05). The type of surgery (mastectomy or WLE) did not affect survival in women with ductal carcinoma-in situ, while women with invasive cancer appeared to survive longer when treated with WLE (P < 0.01). Surgery type was not an independent predictor of overall survival and the survival advantage with WLE did not remain after adjusting for age, implying that the effect on survival had been confounded by the fact that older women tended to undergo mastectomy. Mastectomy remains common among our local women, with further studies being needed to evaluate factors involved in decision-making. Older women and those with significant co-morbidities were more likely to undergo mastectomy and this contributed to an apparent survival advantage following WLE.

## Background

Wide local excision (WLE) was widely accepted as an alternative to total mastectomy after large studies showed that the less radical resection produced superior cosmesis without compromising long-term survival (Fisher et al. 2002; Veronesi et al. 1981). Greater sub-specialisation, coupled with growing affluence and an increase in small screen-detected tumours, has led to more women receiving WLE (Albain et al. 1996). Wide local excision accounts for 60–70 % of surgeries performed in women with early stage breast cancer in the developed countries, where women tend to place greater emphasis on body image and sexuality, are more aware of the available treatment options and are more involved in the decision-making (Agarwal et al. 2014; Lazovich et al. 1999). Neoadjuvant chemotherapy, originally used to downstage inoperable tumours, has also been employed to increase WLE rates (Fisher et al. 1997).

Mastectomy rates have remained high in Singapore and many women continue to choose mastectomy over WLE. A recent study from another local institute reported that 70 % of their patients treated between 2001 and 2010 underwent mastectomy (Sim et al. 2014). Our high mastectomy rates seem contrary to the trend in many other developed countries and have persisted despite growing affluence, high adult literacy rates, well-publicised breast cancer awareness campaigns and a national breast cancer screening program. But although WLE achieves equivalent long-term outcomes through less extensive and disfiguring surgery, it entails the need for post-operative irradiation of the remnant breast and this factors into the choice for WLE. Women with less comprehensive health insurance coverage or who stay a considerable distance away from a facility that offers radiation treatment were reportedly less likely to undergo WLE (Nattinger et al. 1992; Chagpar et al. 2006). Such socioeconomic factors may not influence decision-making in our local setting to the same extent since government healthcare schemes subsidise a significant portion of the treatment costs and accessibility to radiation facilities is not an issue in Singapore. Even so, with our co-payment scheme, WLE and radiation therapy will cost about double that a mastectomy and many women are also deterred by the daily treatment sessions required. From an oncological standpoint, this prevalence of mastectomy matters little since long-term survival following both mastectomy and WLE are similar (Fisher et al. 2002; Veronesi et al. 2002). However, recent reports have suggested that mastectomy is in fact associated with poorer survival among women with Stage I and II breast cancers (Agarwal et al. 2014; Hwang et al. 2013). Given that a significant proportion of our local women who are eligible for WLE opt for mastectomy instead, it would be important to determine whether WLE does indeed confer a survival advantage.

The primary objectives of this study were to evaluate the trend in the types of surgery (mastectomy versus WLE) performed at our institute over a 10-year period and to determine whether the type of surgery affects the clinical outcome, in terms of overall survival and disease recurrence. In addition, we also examined the factors more common among women treated with mastectomy and sought to identify patient and disease factors that may have contributed to the surgery trends over the years.

## Methods

A retrospective review was performed of 2244 women who underwent curative breast cancer surgery, including mastectomy, mastectomy and immediate breast reconstruction (IBR) and wide local excision (WLE), at our institute from 1st January 2001 to 31st December 2010. This study has Ethics Committee approval (DSRB2010/00360). Male patients, those with metastatic disease undergoing palliative mastectomy and those who did not receive treatment at our institute after the initial diagnosis were excluded.

The option of WLE was offered to all eligible women, such as those with small tumours in relation to the breast volume, without contraindications or objections to post-operative radiation therapy; those with multicentric or multifocal tumours, or tumours close to the nipple areolar complex were considered on a case-by-case basis. Some surgeons recommend mastectomy for invasive lobular carcinoma because of the more frequent association with multifocal disease, while others include breast magnetic resonance imaging (MRI) in the pre-operative work-up before proceeding with WLE. Other tumour factors do not affect decision-making. Several other factors, such as advanced age, pre-existing co-morbidities, poor family and social support, however, influence the choice of surgery. Immediate breast reconstruction with an autologous flap was often offered as an option to women below 60 years of age without significant co-morbidities, and was only performed together with a mastectomy. The more complex oncoplastic techniques now practiced in many centres and reconstruction using breast implants were not performed during this period.

Whole breast irradiation was routinely offered to all women after WLE. A total dose of 50 Gy, with an additional boost of 10 Gy to the tumour bed, was administrated in fractions of 2 Gy over a period of 6 weeks, and has been the standard of care since year 2000. In 2010, a hyperfractionated regimen (42.5 Gy in 16 fractions) was also offered, mostly to women with node-negative disease after reports of efficacy (Whelan et al. 2010). Post-mastectomy radiation was recommended in women with tumours larger than 5 cm, pre-menopausal women with nodal involvement and in all women with N2 (4 or more nodes) involvement. The benefit of radiation in post-menopausal women with N1 disease is still pending results of the SUPREMO trial, and was discussed on a case-by-case basis. An additional boost of 10 Gy to the tumour bed was routinely given, and a higher dose of 16 Gy would be considered if there were concerns about residual disease post-resection. A 3-field technique, which included the whole breast or chest wall as well as the supraclavicular fossa, was used when there was nodal involvement, and a 2-field technique was used post-WLE for node-negative disease. Systemic treatment recommendations were in accordance with the current NCCN guidelines; hormonal therapy was recommended for hormone-responsive tumours and trastuzumab for HER2-positive tumours larger than T1b. Chemotherapy, commonly anthracycline and/or taxane-based, was recommended for node-positive disease and for node-negative tumours larger than T1c. Oncotype DX assay was discussed on an individual basis.

Data was collected from the clinical records and included demographic data, clinical presentation, the type of surgery performed and standard pathological and outcome parameters. Surgery type was correlated with patient demographics and standard clinicopathological parameters using the Chi-squared test, *t* test and one-way ANOVA as appropriate. Univariate analyses were performed with GraphPadPrism version 6 (GraphPad software Inc., San Diego, CA, USA). Logistic regression was used to identify independent risk factors associated with mastectomy and disease recurrence, and was carried out using the Stata package release 11.0 (Stata Corporation, 4905 Lakeway Drive, College Station, Texas 77845, USA). A full model was first created to include all potentially important explanatory variables. At each step, the variable with the smallest contribution to the model was removed, until a final backward stepwise model was obtained. Linear regression analysis was applied to study the trends in surgery, patient age, tumour size and disease stage, over the surgical years. Kaplan–Meier survival curves were calculated using death as the endpoint. Overall survival between those treated with mastectomy versus those treated with WLE was compared using a log rank test. Multivariable cox proportional hazard regression modeling was performed to examine the effect of the type of surgery on overall survival after controlling for age, tumour size and ER status, nodal status, disease recurrence and systemic treatment. Adjusted survival curves were then calculated and plotted based on the multivariable cox regression models generated. A 2-tailed P value test was used in all analyses and a P value of less than 0.05 was considered statistically significant.

## Results

Patient and tumour characteristics are detailed in Table 1. A total of 2244 women with operable breast cancer underwent curative surgery at our institute from 2001 to 2010. More than half the women (1312 of 2244, 58 %) were treated with mastectomy, and of these, 146 women (11.1 %) underwent reconstruction at the same setting. Median age at diagnosis was 54 years (ranging from 23 to 94 years of age) and only 8.8 % of women (198 of 2244) were younger than 40 years of age. Three hundred and thirty-four women (14.9 %) were diagnosed with ductal carcinoma-in situ (DCIS), 676 (30.1 %) with Stage I, 762 (34.0 %) with Stage II and 472 (21.0 %) with Stage III disease. Over the median follow-up period of 90 months (ranging from 2 to 167 months), 146 women developed locoregional recurrence, and distant disease was found concurrently or subsequently in 80 of them. Distant disease recurrence in the absence of locoregional disease, developed in 194 women and 338 women died during the follow-up period.

**Table 1:** Correlation analyses of the type of surgery and standard clinicopathological parameters (n = 2244)

	Mastectomy (n = 1312)	Wide excision (n = 932)	*P* value
Median age	55 (23–94)	51 (24–90)	<0.01
Menstrual status			0.01
Pre-menopausal	945	446	
Post-menopausal	815	482	
Ethnicity			0.06
Chinese	1064	748	
Malay	120	78	
Indians	59	65	
Others	69	41	
Presentation			<0.01
Screen detected	219	324	
Symptomatic	1067	582	
Median clinical tumour size (cm)	3.0 (0.5–20)	2.0 (0.5–6.0)	<0.01
Clinically palpable axillary nodes			<0.01
Yes	149	26	
No	1141	896	
Pre-operative diagnosis			<0.01
Invasive CA	1092	673	
DCIS	218	252	
Atypia	2	7	
Neoadjuvant chemotherapy			<0.01
Yes	93	13	
No	1219	919	
Median pathological tumour size (mm)	25 (1–210)	16 (1–70)	<0.01
Tumour histology			<0.01
DCIS	165	221	
IDC	1029	655	
ILC	76	22	
Medullary CA	3	5	
Mucinous CA	16	13	
Papillary CA	3	7	
Others	19	5	
Tumour grade			0.09
1	169	128	
2	501	313	
3	451	265	
ER status			<0.01
Positive	847	621	
Negative	394	209	
PR status			0.02
Positive	618	456	
Negative	621	373	
Nodal status			<0.01
Positive	602	299	
Negative	575	502	
Disease stage			<0.01
DCIS	135	199	
I	311	365	
II	473	289	
III	393	79	
ASA score^a^			<0.01
1	81	62	
2	454	286	
3	87	28	

^a^Information available only from 2006 onwards

**Fig. 1:**
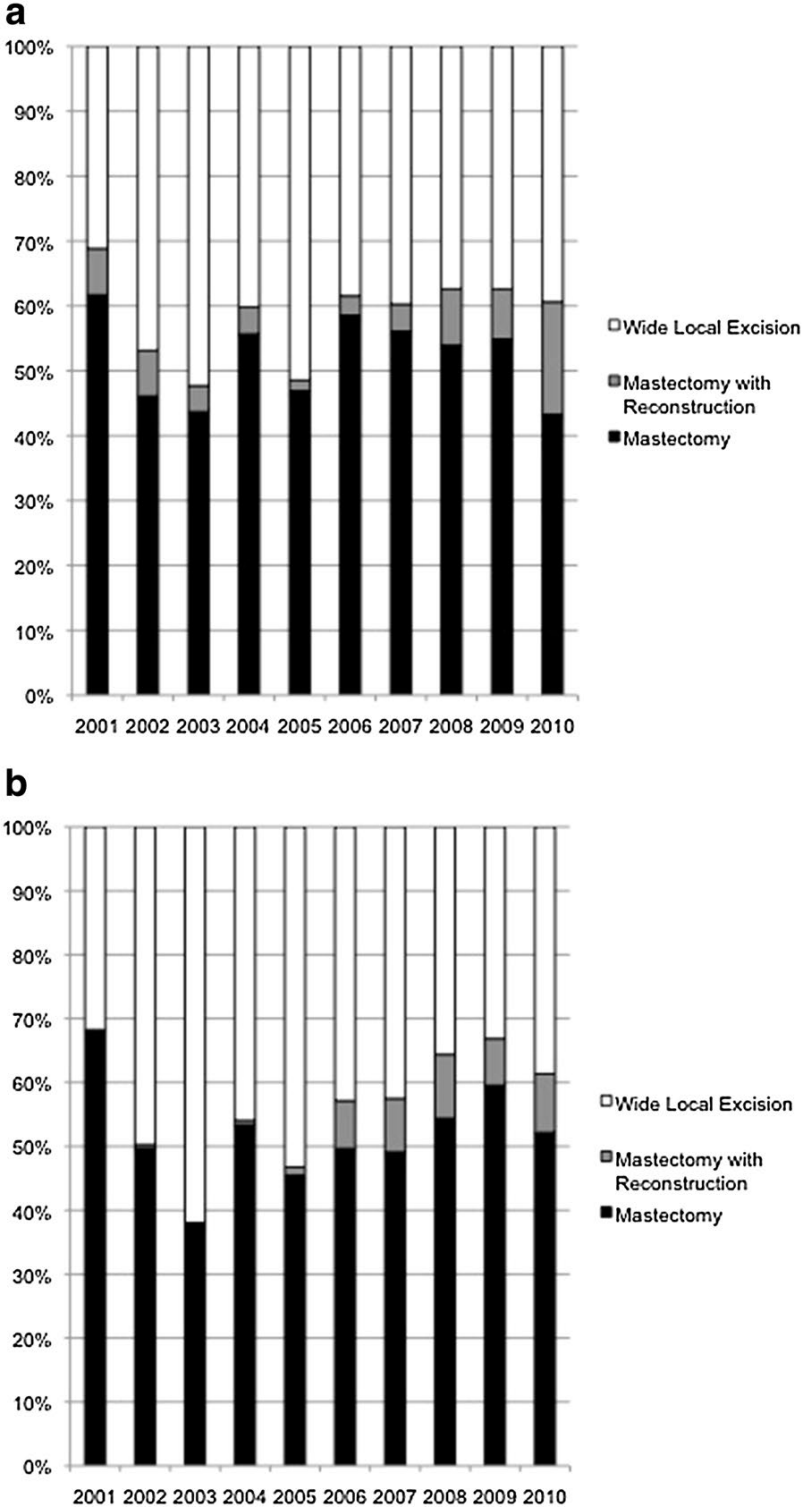
**a** The proportion of surgeries performed at our institute by surgical year. **b** The proportion of surgeries performed in women with Stage I and II cancers at our institute by surgical year

While the absolute numbers of surgeries performed at our institute increased steadily over the 10-year study period, the proportion of mastectomies relative to WLE did not change significantly with the surgical years (Fig. 1a). There was an initial drop in the rate of mastectomies (without reconstruction) from 62 % in 2001 to 44 % in 2003, coinciding with the implementation of nationwide breast cancer screening in 2002, but thereafter, the mastectomy rate varied from between 43 to 59 % and averaged at 53 % over the subsequent years (P = 0.54, β = 0.49 ± 0.76). More than half the women with Stage I and II cancers underwent mastectomy and there was no declining trend over the years (P = 0.48, β = 0.73 ± 0.99) (Fig. 1b). This could not be attributed to more women opting for IBR, which accounted for only about 7 % of all mastectomies performed (P = 0.14, β = 0.73 ± 0.45). We did, however, observe a more than two-fold increase in IBR rates in the last 2 years, from 7 to 17 %; this increase was largely among women with Stage III disease (Fig. 1a, b).

Age and existing co-morbidities correlated strongly with the type of surgery received. Older women (P < 0.01) with major co-morbidities (P < 0.01), implied by higher ASA scores were more likely to undergo mastectomy, as were those who had presented with symptoms (rather than with screen-detected tumours) (P < 0.01, OR 2.71, 95 % CI 2.22–3.31) (Table 1). Consistent with tumour size being a major consideration for WLE, women treated with mastectomy had larger tumours (P < 0.01, OR 10.48, 95 % CI 0.05–0.17) and were more likely to have a pre-operative diagnosis of invasive carcinoma (P < 0.01, OR 1.88, 95 % CI 1.53–2.30), particularly that of invasive lobular carcinoma (P < 0.01, OR 2.19, 95 % CI 1.35–3.56). Women who had received neoadjuvant chemotherapy were more also likely to undergo mastectomy, regardless of the degree of tumour response (P < 0.01, OR 5.39, 95 % CI 3.00–9.70). The observed association with nodal involvement, negative tumour ER status and tumour grade was due to a correlation with larger tumours (P < 0.01) (Table 2). Only the age at diagnosis, mode of presentation and disease stage remained independently associated mastectomy on multivariate analysis (P < 0.05) (Table 3).

**Table 2:** Correlation of tumour size with tumour grade and ER status (n = 2757)

Parameter	Median tumour size (mm)	P value
Nodal status		<0.01
Positive	28 (1–210)	
Negative	18 (1–125)	
Tumour grade		<0.01
1	15 (1–140)	
2	20 (1–150)	
3	25 (1–210)	
ER status		<0.01
Positive	20 (1–140)	
Negative	25 (1–210)

**Table 3:** Logistic regression model of the type of surgery received for standard clinicopathological parameters (n = 1042)

Parameter	Odds ratio	SE	P value	95 % CI
Age at diagnosis	1.04	0.01	<0.01	1.02–1.05
ASA score	1.14	0.15	0.31	0.88–1.49
Clinical presentation^a^	2.07	0.34	<0.01	1.50–2.84
Clinical tumour size	7.50	4.62	<0.01	2.24–25.11
Clinically palpable nodes	8.19	5.13	<0.01	2.41–27.92
Pre-operative biopsy	1.19	0.21	0.33	0.84–1.67
ER status	0.78	0.12	0.11	0.57–1.06
Neoadjuvant chemotherapy	1.68	0.74	0.24	0.71–3.98

^a^Refers to whether tumours were symptomatic at presentation or were screen-detected

**Fig. 2:**
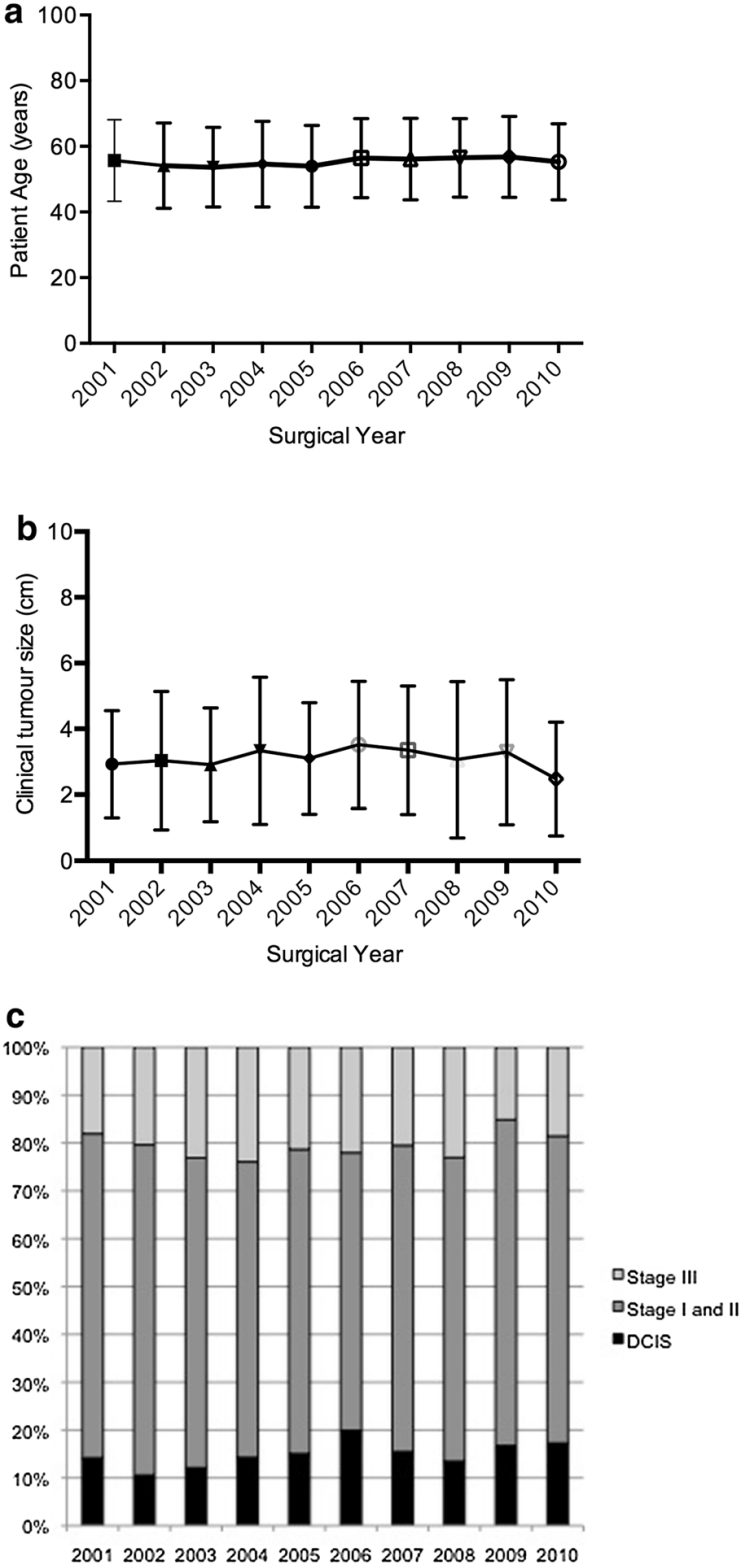
**a** Mean patient age (years) by surgical year. **b** Mean clinical tumour size (cm) by surgical year. **c** Disease stage at presentation by surgical year

We next examined the contribution of patient age and disease stage to the persistently high mastectomy rates. Mean age at diagnosis ranged from between 51 to 58 years of age (median age from 51 to 59 years), and although there seemed to be a gradual increase in the age at diagnosis over the years, this was not statistically significant (P = 0.08, β = 0.22 ± 0.11) (Fig. 2a). Women older than 70 years of age consistently accounted for 12–19 % of cancers diagnosed each year (P = 0.81). Likewise, women younger than 40 years also accounted for <10 % of the cancers diagnosed each year. Tumour size remained similar over the years as well (P = 0.85, β = −0.01 ± 0.03 and P = 0.58, β = −0.23 ± 0.39, respectively) (Fig. 2b); mean tumour size at presentation ranged from 2.47 ± 0.12 to 3.52 ± 1.93 cm and median size was 2.75 cm (ranging from 1.0 to 20.0 cm). Only about 8 % of tumours diagnosed each year were larger than 5 cm. Mastectomy was performed in 510 of the 1263 women (40.4 %) younger than 70 years of age with tumours smaller than 2 cm. There was no significant change in the disease stage at presentation (P = 0.07, β = 0.51 ± 0.24, P = 0.52, β = −0.27 ± 0.41 and P = 0.51, β = −0.24 ± 0.35 for DCIS, Stage I and II and Stage III disease, respectively) (Fig. 2c). Close to two-thirds of women presented with Stage I and II disease, up to 19 % presented with DCIS and about 20 % presented with advanced disease.

**Fig. 3:**
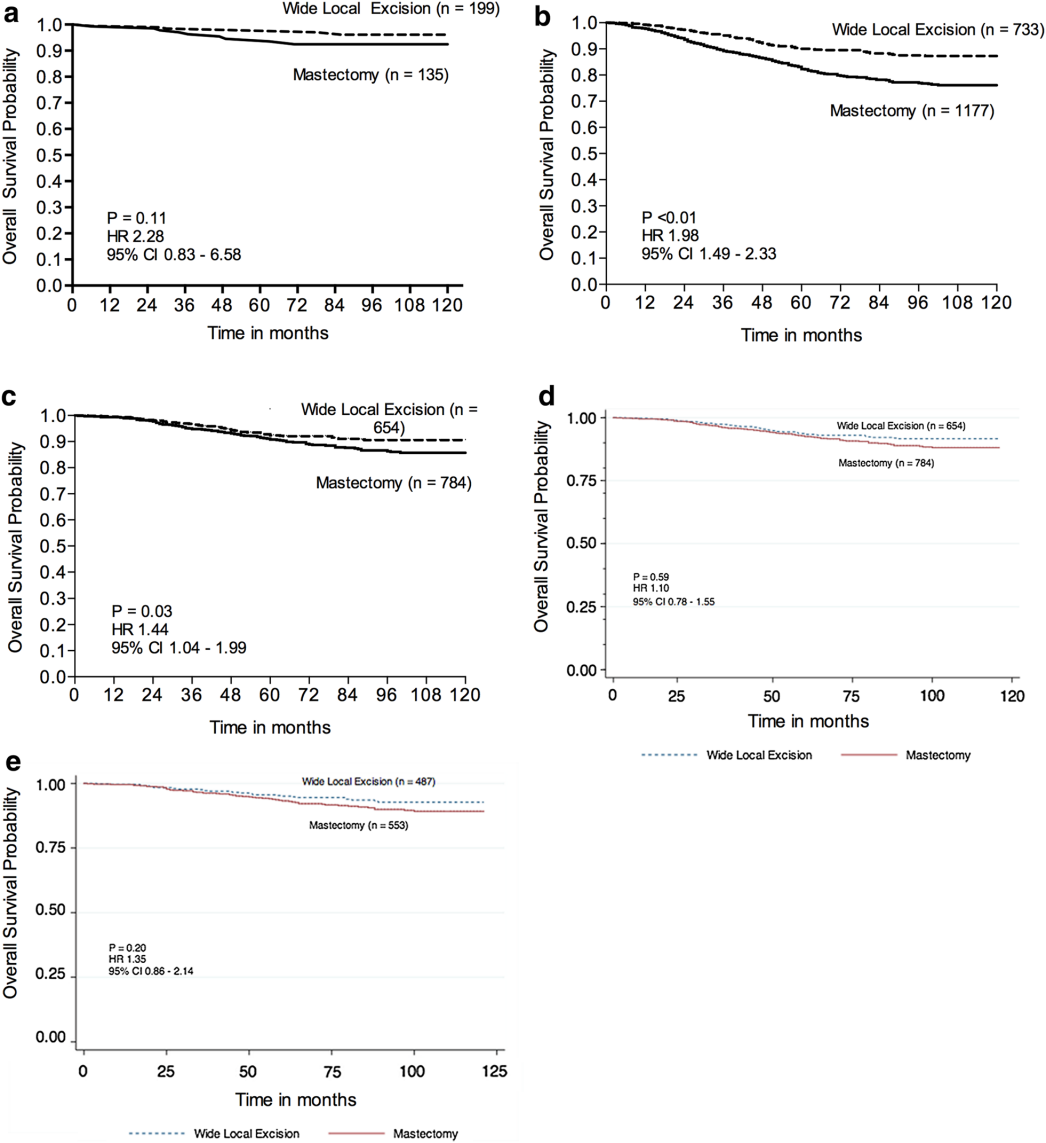
**a** Kaplan and Meier curves of 10-year overall survival stratifying patients with DCIS by the type of surgery received (n = 334). **b** Kaplan and Meier curves of 10-year survival stratifying patients with Stage I to III disease by the type of surgery received (n = 1910). **c** Kaplan and Meier curves of 10-year overall survival stratifying patients with Stage I and II disease by the type of surgery received (n = 1438). **d** Survival curves of 10-year survival stratifying patients with Stage I and II disease by the type of surgery received, adjusted for age at time of diagnosis (n = 1429). **e** Survival curves of 10-year survival stratifying patients with node negative Stage I and II disease by the type of surgery received, adjusted for age at time of diagnosis (n = 1040)

Next, we evaluated the impact of the type of surgery on clinical outcome. Among women with DCIS, overall survival was similar regardless of whether a mastectomy or WLE was performed (Fig. 3a). Wide local excision appeared to confer a statistically significant survival advantage in women with invasive cancer (P < 0.01, HR 1.98, 95 % CI 1.49–2.33) (Fig. 3b), and this was significant even when Stage III disease was excluded (P = 0.03, HR 1.44, 95 % CI 1.04–1.99) (Fig. 3c). Surgery type was no longer significant when included in a multivariate model (P = 0.95) (Table 4), where age, tumour size and disease recurrence emerged as independent predictors of survival (P < 0.05), and the survival difference was no longer present after adjusting for age at diagnosis (Fig. 3d). We separately analysed node-negative Stage I and II cancers and again did not find a difference between survival after a mastectomy or a WLE (Fig. 3e). Wide local excision was associated with a higher local recurrence rate (P = 0.01, OR 1.89, 95 % CI 0.33–0.86) (Table 5a) but not distant recurrence (P = 0.44), which was instead predicted by unfavourable tumour characteristics such as larger tumour size, nodal involvement, ER-negativity, and local recurrence (P < 0.05) (Table 5b).

**Table 4:** Multivariate analysis cox regression model of overall survival for mastectomy and standard clinicopathological parameters in patients diagnosed between 2001 and 2010 (n = 1382)

Parameter	Hazard ratio	SE	P value	95 % CI
Type of surgery received	0.98	0.25	0.95	0.59–1.63
Age at diagnosis	1.08	0.01	<0.01	1.06–1.10
Tumour size	1.02	0.01	0.04	1.00–1.03
ER status	0.64	0.16	0.07	0.39–1.03
Nodal status	1.30	0.33	0.31	0.79–2.15
Disease recurrence	57.29	16.60	<0.01	32.47–101.08
Systemic treatment omitted^a^	0.73	0.33	0.49	0.30–1.78

^a^Chemotherapy, hormonal therapy (and targeted therapy from 2006 onwards)

**Table 5:** Logistic regression model of (a) local recurrence and (b) distant recurrence for type of surgery received and standard clinicopathological parameters, in patients diagnosed between 2001 and 2010 (n = 1382)

Parameter	Odds ratio	SE	P value	95 % CI
(a)				
Type of surgery	0.48	0.13	0.01	0.29–0.80
Age at diagnosis	0.99	0.01	0.49	0.97–1.03
Tumour size	1.01	0.01	0.14	0.10–1.03
ER status	0.45	0.12	<0.01	0.27–0.76
Nodal status	1.49	0.41	0.15	0.87–2.57
Systemic therapy omitted^a^	6.02	2.00	<0.01	3.13–11.55
(b)				
Type of surgery	1.20	0.28	0.44	0.76–1.90
Age at diagnosis	1.00	0.01	0.78	0.99–1.02
Tumour size	1.03	0.01	<0.01	1.01–1.04
ER status	0.63	0.14	0.04	0.40–0.99
Nodal status	1.71	0.39	0.02	1.09–2.68
Local recurrence	13.15	3.68	<0.01	7.60–22.76
Systemic therapy omitted^a^	1.71	0.66	0.17	0.80–3.63

^a^Systemic therapy includes chemotherapy and hormonal therapy

## Discussion

In our study, we observed that older women tended to undergo mastectomy; 19 % of those who had a mastectomy were older than 70 years of age as compared to 8 % of those who had a WLE. We were unable to conclude whether this was a preference on the part of the patient or the surgeon but from our experience, older women often prioritise a shorter treatment course over post-operative cosmesis and would rather avoid a repeat operation and daily post-operative radiation sessions. Financial costs may also be a contributing factor. Surgeons may also be more inclined towards mastectomy in older women, particularly those with significant co-morbidities who could have a higher risk of potential morbidity from additional surgery and radiation treatments. Even so, as many as 40 % of our women younger than 70 years of age with tumours smaller than 2 cm underwent mastectomy, instead of WLE. This may seem a relatively high proportion, but Asian women generally have smaller breast volumes and a 2 cm tumour may be too large or too close to the nipple areolar complex for WLE. Median tumour size in our study was 2.1 cm, and was larger than most tumours in the studies where WLE was found superior to mastectomy (Agarwal et al. 2014; Hwang et al. 2013). Furthermore, many women opt for mastectomy rather than take up neoadjuvant chemotherapy to downsize a tumour deemed too large for breast conservation. The tendency for women with symptoms to undergo mastectomy may reflect differences in the attitudes and psyche of women presenting to the clinics. Women with symptoms often seek medical attention because they suspect a cancer and often seem to assume that mastectomy would be offered once the diagnosis is confirmed. On the other hand, women attending breast screening are often unprepared for a cancer diagnosis and tend to opt for less extensive surgery. Regardless, our study suggests that many women eligible for WLE undergo a mastectomy instead.

Some centres in the West have reported an increase in mastectomies in recent years, citing the more frequent use of pre-operative magnetic resonance imaging (MRI) that has increased the detection of additional occult tumour foci elsewhere in the breast (Katipamula et al. 2009). This was unlikely to have contributed to our high mastectomy rates, since breast MRI is limited to women with invasive lobular carcinomas who are keen for WLE. Growing preference for breast reconstruction may also have contributed to this increase, particularly among younger women who find the more natural appearing post-operative body image especially appealing (Alderman et al. 2008). Furthermore, though breast conservation is not contraindicated in young women, surgeons have been known to recommend mastectomy more strongly because of reportedly higher local failure rates after WLE (Voogd et al. 2001; de Bock et al. 2006). Reconstruction accounted for less than a quarter of our cases and had not increased much except in the last 2 years, making it unlikely to have accounted for the persistently high mastectomy rates. A more advanced age and disease stage at diagnosis were also unlikely contributing factors. The average age at diagnosis had not increased significantly over the years and was in fact similar to that reported in the Surveillance, Epidemiology, and End Results data. Significantly, the numbers of women older than 70 years or younger than 40 years, who more often received mastectomy, had not increase. Similar numbers of women remained eligible for WLE and <10 % of tumours were staged as T3 or T4 at presentation.

In keeping with published reports, local recurrence was two-fold higher following WLE, even with standard radiation doses (Darby et al. 2011). Despite a correlation between local recurrence and distant relapse, we did not find WLE to adversely affect long-term survival, reaffirming findings from previous studies (Fisher et al. 2002; Veronesi et al. 1981; Blichert-Toft et al. 2008). Our observation that 70 % of distant recurrences occurred in the absence of locoregional disease, while only 45 % of those with locoregional recurrence developed systemic disease, supports the postulation that distant disease relapse is not necessarily a direct consequence of untreated locoregional disease (Le et al. 2002). In fact, locoregional recurrences are often isolated and amenable to local therapy, and thus may not affect survival (Schmoor et al. 2000). Rather, distant relapse is more likely a manifestation of inherent tumour aggressiveness, as evident from the association with unfavourable disease factors such as larger tumour size, nodal involvement and ER negativity.

Our findings are in direct contrast to two recent studies that reported a survival advantage with WLE, even after controlling for established prognostic factors such as tumour size and nodal status (Agarwal et al. 2014; Hwang et al. 2013). We, too, observed a similar survival benefit among women who received WLE, but this survival difference was no longer present after adjusting for patient age. This implied that the apparent survival difference was due to inherent differences in the women included in both groups, and was because women who underwent mastectomy tended to be older and with more co-morbidities. Hwang and colleagues found WLE to confer a survival advantage even after excluding women who received post-mastectomy irradiation (more advanced disease), but this could have been confounded by more women with node-negative disease undergoing WLE (Hwang et al. 2013). We therefore specifically analysed women with node-negative Stage I and II disease, who would have either received no radiation after mastectomy or a 2-field radiation technique, which did not include the level II and III nodes, after WLE. Again, we did not observe any survival difference between WLE and mastectomy after adjusting for age, and this strengthens our hypothesis that the apparent survival advantage of WLE was primarily due to the women being younger and with less co-morbidities. This is consistent with our observation that older women, who also tend to have greater co-morbidities (higher ASA scores), were more likely to receive mastectomy. Mastectomy is often seen as the better option in such poor-risk patients since it avoids the need for a repeat surgery and daily radiation treatments. This was in fact alluded to in one of the two reports of WLE superiority, which showed that women treated with WLE had reduced mortality from cardiovascular, cerebrovascular and chronic respiratory causes (Hwang et al. 2013). It seems unlikely that the type of operation itself would affect mortality, since the surgical risks involved are similar and there is no convincing evidence that mastectomy causes greater physiological disturbances or long-term health effects. Post-operative recovery is also relatively similar and both surgeries are routinely performed as ambulatory procedures at our institute (Ng et al. 2014). On the contrary, post-WLE irradiation to the left breast may even increase mortality from late onset cardiotoxicity (Darby et al. 2013).

## Conclusions

Mastectomy rates at our institute have remained high over the last 10 years, and cannot be attributed to an increased uptake of immediate breast reconstruction, or to more advanced age or disease at presentation. Having observed that many women eligible for WLE undergo mastectomy instead, further studies would provide useful insight into the factors and dynamics that influence the decision-making process. Importantly, we found that overall survival was similar regardless of the type of surgery performed, and that the apparent survival advantage of WLE could be attributed to younger and good-risk patients receiving WLE.
